# Effectiveness and feasibility of 5G-based remote interactive ultrasound training in critical care

**DOI:** 10.1186/s12909-024-05485-0

**Published:** 2024-05-08

**Authors:** Peng Shen, Youzhong An, Chenxiao Hao, Jie Lyu, Huiying Zhao

**Affiliations:** Department of Critical Care Medicine, Peking University People’s Hospital, No. 11 Xizhimen South Street, Xicheng District, Beijing, 100044 China

**Keywords:** 5G, Ultrasound training, Remote critical care ultrasound, Simulation training

## Abstract

**Background:**

Ultrasound has widely used in various medical fields related to critical care. While online and offline ultrasound trainings are faced by certain challenges, remote ultrasound based on the 5G cloud platform has been gradually adopted in many clinics. However, no study has used the 5G remote ultrasound cloud platform operating system for standardized critical care ultrasound training. This study aimed to evaluate the feasibility and effectiveness of 5G-based remote interactive ultrasound training for standardized diagnosis and treatment in critical care settings.

**Methods:**

A 5G-based remote interactive ultrasound training system was constructed, and the course was piloted among critical care physicians. From July 2022 to July 2023, 90 critical care physicians from multiple off-site locations were enrolled and randomly divided into experimental and control groups. The 45 physicians in the experimental group were trained using the 5G-based remote interactive ultrasound training system, while the other 45 in the control group were taught using theoretical online videos. The theoretical and practical ultrasonic capabilities of both groups were evaluated before and after the training sessions, and their levels of satisfaction with the training were assessed as well.

**Results:**

The total assessment scores for all of the physicians were markedly higher following the training (80.7 ± 11.9) compared to before (42.1 ± 13.4) by a statistically significant margin (*P* < 0.001). Before participating in the training, the experimental group scored 42.2 ± 12.5 in the critical care ultrasound competency, and the control group scored 41.9 ± 14.3—indicating no significant differences in their assessment scores (*P* = 0.907). After participating in the training, the experimental group’s assessment scores were 88.4 ± 6.7, which were significantly higher than those of the control group (72.9 ± 10.8; *P* < 0.001). The satisfaction score of the experimental group was 42.6 ± 2.3, which was also significantly higher than that of the control group (34.7 ± 3.1, *P* < 0.001).

**Conclusion:**

The 5G-based remote interactive ultrasound training system was well-received and effective for critical care. These findings warrant its further promotion and application.

## Background

In recent years, ultrasound has gradually become a new type of “stethoscope” for intensivists and has been widely used in various medical fields related to critical care. However, ultrasound differs from radiography and computed tomography examinations, and its diagnostic accuracy is closely related to the specific ultrasound skills of critical care physicians. The use of non-standardized ultrasound methods by doctors without formal training is likely to lead to biased diagnostic results, thereby affecting the treatment of critically ill patients [1, 2]. Online ultrasound training can impart theoretical knowledge of critical care ultrasound but cannot provide timely counseling and corrections for the trainee’s operation and image acquisition details. Offline ultrasound training is often subject to venue and time constraints that can increase learning costs for nonlocal trainees. With the emergence and popularization of 5G technology and cloud computing, which solve problems related to network delays, storage, and information sharing, remote ultrasound based on the 5G cloud platform has also been gradually adopted in many clinics [3, 4]. To the best of our knowledge, this study is the first attempt to use the 5G remote ultrasound cloud platform operating system for standardized critical care ultrasound training. This study aimed to provide remote online guidance to trainees regarding best practices for ultrasound probe operation techniques, image acquisition sites, and image quality optimization. We then evaluated the feasibility and effectiveness of this system in terms of physician competencies and satisfaction.

## Methods

### Course content design

The study began with the trainees themselves, as they took the initiative to help design and construct the content of the 5G critical care ultrasound training program, and we repeatedly solicited their opinions to optimize its usefulness; 12 critical care physicians from our hospital and 18 critical care physicians from other nonlocal hospitals were recruited for this study between February and May of 2022. Under the guidance of the critical care ultrasound teaching team (two associate chief physicians and three attending physicians), the trainees were allowed to combine the problems they encountered in their clinical work, review the information, and discuss with one another to design the content that they needed, which included: (1) the basic principles and fundamentals of critical care ultrasound; (2) standardized training in cardiac critical care ultrasound; (3) standardized training in pulmonary critical care ultrasound; (4) standardized training in abdominal critical care ultrasound; (5) standardized training in neurological critical care ultrasound; (6) standardized training in ultrasound-guided simulated puncture tube placement.

The ultrasound simulator system of our clinical competence training center was then used to simulate ultrasound imaging for standard normal adults and various types of critical care patients with related diseases, according to the final content of the course. The 5G remote visualization cloud platform system interfaced with the ultrasound simulator and bedside ultrasonography systems. This was done so that data transmission could be used to help the nonlocal trainees to be instructed in specific ultrasound probe operation techniques, proper selection of image acquisition sites, and optimization of ultrasound image quality. The 5G remote blue membrane ultrasound intervention simulation system was also used to remotely guide the remote trainees to virtually complete the arterial vein simulation puncture operation by hand. Finally, a complete set of 5G-based remote interactive ultrasound training and teaching systems covering common scenarios in clinical practice was established.

### Study participants

Ninety nonlocal intensive care unit physicians were enrolled between June 2022 and June 2023. They included 13 chief/associate chief physicians, 32 attending physicians, and 45 residents. This study was approved by the Ethics Committee of Peking University People’s Hospital, and all participants provided informed consent to participate. This was a randomized controlled study in which the 90 participants were divided into experimental and control groups of 45 members each, using the random number table method.

### Program implementation

**Fig. 1:**
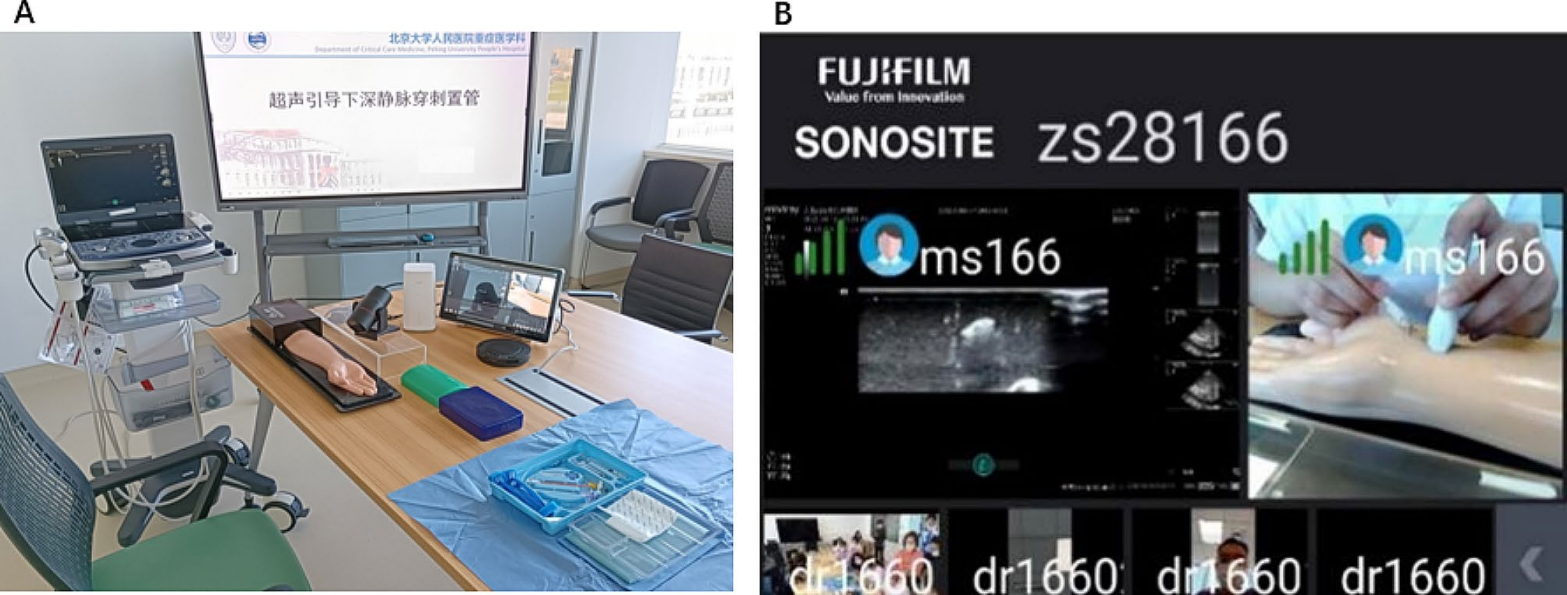
Interactive 5G remote critical care ultrasound training device. **A**: 5G remote critical care ultrasound visualization cloud platform device connection; **B**: 5G interconnected multi-platform operator image transmission and debugging

**Fig. 2:**
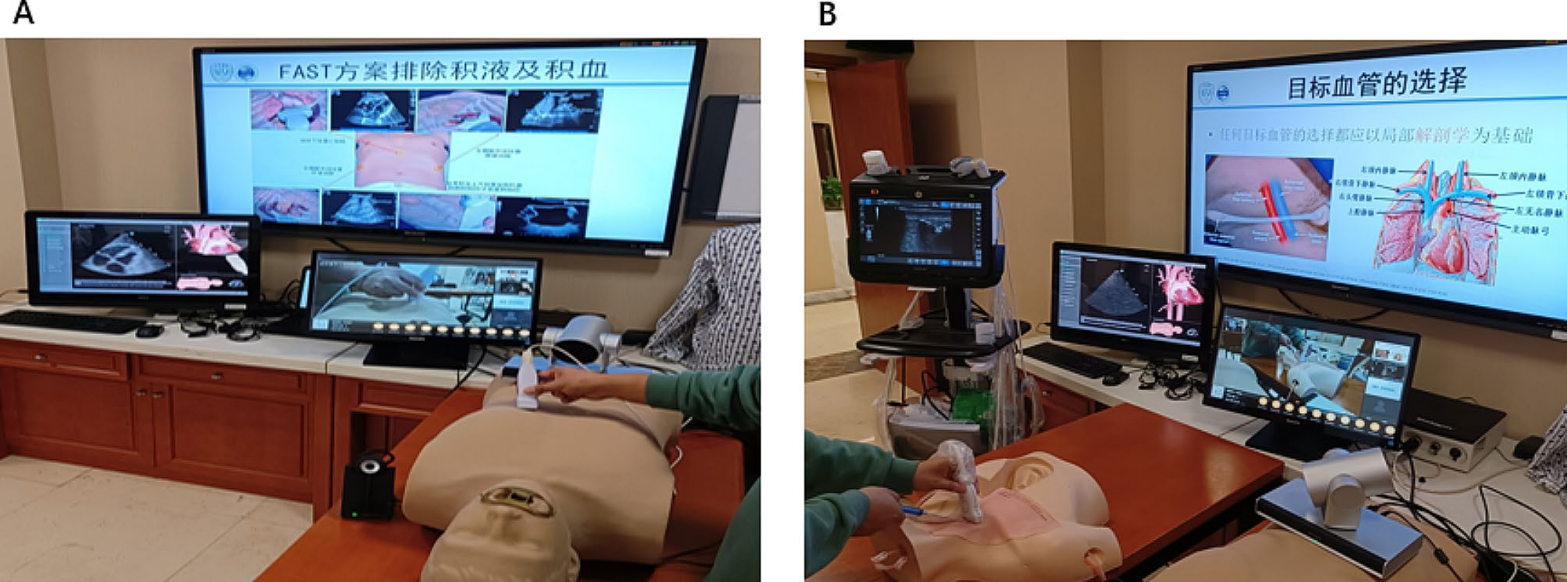
Demonstration of 5G-based remote interactive ultrasound training. **A**: The teacher conducts online theoretical lectures and demonstrates the operation of various systems of critical care ultrasound simulators with the help of a 5G remote cloud platform system. **B**: The teacher conducts an online demonstration of simulated ultrasound puncture with the help of a 5G remote cloud platform system

The experimental group used the 5G-based remote interactive ultrasound training system. Before training, they docked with the remote training trainee units configured with bedside ultrasound, mailed the 5G remote cloud platform module and blue membrane ultrasound intervention model to one another’s training sites in advance, and remotely instructed one another regarding debugging and docking the equipment. Concurrently, the 5G remote cloud platform module was docked and debugged with our ultrasound simulator system at our hospital’s Clinical Competence Training Center, and the specific processes and precautions for teaching the program were communicated in advance **(**Fig. 1**)**. Remote theoretical and practical simulations were then conducted, and remote trainees were instructed to complete the practical exercises and assessments in each system **(**Fig. 2**)**. The contents were as follows: (1) basic principles and knowledge regarding critical care ultrasound; (2) cardiac critical care ultrasound theoretical lectures, an accompanying simulator-based teaching demonstration, and practical remote guidance; (3) lung critical care ultrasound theoretical lectures, an accompanying simulator-based teaching demonstration, and practical remote guidance; (4) abdominal critical care ultrasound theoretical lectures, an accompanying simulator-based teaching demonstration, and practical remote guidance; (5) neurological critical care ultrasound theoretical lectures, an accompanying simulator-based teaching demonstration, and practical remote guidance; (6) ultrasound-guided simulated puncture placement theory, an accompanying simulator-based teaching demonstration, and hands-on remote instruction on the interventional model. Conversely, the participants in the control group were taught theory through traditional remote video online conferences.

### Evaluation of training effectiveness

Theoretical and operational assessments were conducted for the two groups of critical care ultrasound trainees before and after the training sessions to compare the effectiveness of the two methods. The combined theoretical and practical examination was scored on a total of 100 points, with the scoring breakdown including: (1) a theoretical examination of the basic principles of critical care ultrasound, commonly used modalities, and the significance of relevant parameters (25 points); (2) a theoretical examination of critical care ultrasound of the heart, accompanied by the practical simulated acquisition and interpretation of the basic views (15 points); (3) a theoretical examination of critical care ultrasound of the lungs and diaphragm, accompanied by the practical simulated acquisition and interpretation of the basic views (15 points); (4) a theoretical assessment of abdominal critical care ultrasound, accompanied by the practical simulated acquisition and interpretation of the basic views (15 points); (5) a theoretical assessment of neurological critical care ultrasound, accompanied by the practical simulated acquisition and interpretation of the basic views (15 points); (6) a theoretical assessment of ultrasound-guided simulated puncture and tube placement, accompanied by practical exercises (15 points).

To better evaluate the effectiveness of the teaching from the perspectives of the trainees, we asked the trainees to rate their level of satisfaction with what they learned 1 month after the end of the training (to allow sufficient time for the trainees to experience the real-world value of the training in their daily work).The results of the two groups were compared. The satisfaction evaluation contained five items, each scoring 1–10, for a maximum of 50 points. These items included: (1) increased theoretical knowledge of critical care ultrasound; (2) improved practical ability to perform critical care ultrasound; (3) whether the application of critical care ultrasound has improved the emergency response in the trainee’s workplace; (4) whether the application of critical care ultrasound has reduced errors in the trainee’s clinical work; (5) whether the critical care ultrasound training increased the self-confidence of the trainee’s clinical work.

### Statistical analysis

SPSS 22.0 statistical software (SPSS Inc., Chicago, IL, USA) was used to analyze the data. Measurement information is expressed as means ± standard deviations, and the data were compared using both paired- and independent-samples Student’s t-tests. Count information is expressed as the percentage composition ratio, and comparisons for count data were made using the χ^2^ test. *P* < 0.05 was considered statistically significant.

## Results

Overall, 13 chief/associate chief physicians, 32 attending physicians, and 45 residents participated in the study. In the experimental group, there were 6(13.3%) chief/associate chief physicians, 17 (37.8%) attending physicians, and 22 (48.9%) residents. In the control group, there were 7(15.6%) chief/associate chief physicians, 15 (33.3%) attending physicians, and 23 (51.1%) residents. Therefore, there was no statistical difference in the medical titles of trainees between the two groups (*P* = 0.894). In the experimental group, the pre-training scores of theoretical knowledge and practical operation were 20.9 ± 6.3 and 21.3 ± 7.2, respectively. In the control group, the pre-training scores of theoretical knowledge and practical operation were 21.7 ± 7.7 and 20.2 ± 7.1, respectively. The pre-training total scores were also not significantly different between the two groups, and the homogeneity was comparable, as shown in Table 1.

**Table 1:** Comparison of the general characteristics of the two groups

	Experimental group *n* = 45	Control group *n* = 45	*P*
Men, cases (%)	24 (53.3)	22 (48.9)	0.673
Composition of personnel, cases (%)			0.894
Chief/Associate Chief Physician	6(13.3)	7 (15.6)	
Attending Physician	17 (37.8)	15 (33.3)	
Resident	22 (48.9)	23 (51.1)	
Pre-training assessment results	42.2 ± 12.5	41.9 ± 14.3	0.907

### Comparison of assessment scores and satisfaction ratings between the two groups after the training

After training, the theoretical knowledge score was 43.7 ± 4.8, the practical operation score was 44.8 ± 3.0, and the total score was 88.4 ± 6.7 in the experimental group. In the control group, the theoretical knowledge score was 37.1 ± 7.0, the practical operation score was 35.8 ± 4.6, and the total score was 72.9 ± 10.8 after training. The experimental group scored significantly higher than the control group in terms of critical care ultrasound theory assessment results, ultrasound practical operation assessment results, and total assessment results. Moreover, the theoretical knowledge score, the practical operation score, and the total score of the experimental group and the control group were statistically different (*P* < 0.001), as shown in Table 2. The training satisfaction level of the experimental group was also significantly higher than that of the control group in terms of increasing the level of critical care ultrasound theoretical knowledge, improving practical ultrasound operational ability, improving clinical emergency response ability, reducing errors in clinical work, and increasing confidence of the trainees. Moreover, there were significant statistical differences between the experimental group and the control group in various satisfaction survey scores (*P* < 0.001), as is shown in Table 2.

**Table 2:** Comparison of assessment scores and satisfaction between the two groups after the training

	Experimental group *n* = 45	Control group *n* = 45	*P*
Theoretical assessment results	43.7 ± 4.8	37.1 ± 7.0	< 0.001
Operational assessment results	44.8 ± 3.0	35.8 ± 4.6	< 0.001
Overall assessment results	88.4 ± 6.7	72.9 ± 10.8	< 0.001
Enhancement of theoretical knowledge	8.1 ± 1.2	7.7 ± 0.9	< 0.001
Improvement of ultrasound operation capability	9.1 ± 0.8	5.9 ± 1.3	< 0.001
Improvement of emergency response capacity	8.6 ± 1.1	6.7 ± 1.2	< 0.001
Reducing clinical errors	8.2 ± 0.7	7.4 ± 0.8	< 0.001
Enhancing self-confidence	8.6 ± 0.7	7.5 ± 0.8	< 0.001
Overall satisfaction with training	42.6 ± 2.3	34.7 ± 3.1	< 0.001

## Discussion

In this study, we created a 5G-based remote simulation and practice critical care system for ultrasound visualization training and teaching, including clinical integration and easy-to-understand images. The effectiveness and appeal of this system were verified among remote off-site trainees. This training and teaching system enables clinicians to master the clinical skills of critical care ultrasound and thus ensure higher degrees of medical safety associated with these procedures.

With the recent advancement of ultrasound technology and the development of portable and miniaturized ultrasound systems, bedside ultrasound is gradually becoming a new type of “stethoscope” for intensivists. Currently, it is widely used in various medical contexts, including cardiopulmonary function evaluation, multiple injury exclusion, and auxiliary interventional operations [5–7]. The intensive care and emergency departments of primary hospitals are actively introducing bedside ultrasound and are gradually applying it to the treatment of critically ill patients [8–10]. However, because ultrasound examination is different from X-ray or computed tomography examination, there are strict requirements and standards for the operator to choose the correct site for ultrasound image acquisition, the specific direction and tilt angle of the probe, and the optimization of the quality of the resultant images under different conditions [11, 12]. Unstandardized ultrasound operation methods and substandard ultrasound image data acquisition are likely to lead to biased diagnostic results, thus affecting the treatment of critically ill patients [13–15]. Therefore, standardized training in critical care ultrasonography is essential for critical care physicians.

Ordinary online training courses cannot provide timely counseling and corrections for specific operational techniques and image acquisition details; thus, trainees often report unsatisfactory training. To address the shortcomings of online training, we organized offline ultrasound training courses to provide theoretical knowledge and practical instructions to critical care ultrasound trainees. However, offline teaching modes are often limited by space and time, and using a physical training space can incur additional rental costs. Particularly for nonlocal students, this kind of offline training can often delay their normal work in their home units and pose additional transportation and accommodation problems. All of this can also make the cost of the training excessively high. However, with the emergence and popularization of 5G technology and cloud computing, which solve problems related to network delays, storage, and difficulties in sharing information, remote ultrasound based on 5G cloud platforms is gradually being used in clinical applications [3, 16, 17]. This studyuseda5G remote visualization technology training system to provide trainees with standardized critical care ultrasound imaging training. This training system allowed the trainees to acquire theoretical knowledge related to critical care ultrasound and observe the specific ultrasound practices of the instructor performing clinical work. During the course, the instructor used ultrasound simulators to show ultrasound images of typical cases and analyze their clinical diagnoses and treatment options. During the practice stage, the instructor could also use the 5G remote platform to evaluate the student’s understanding of the course and their practical ultrasound operation abilities. With the help of 5G remote visualization technology, the instructor guided the trainees regarding specific ultrasound probe operation, image acquisition site selection, ultrasound image quality optimization, and other aspects to help them to obtain better ultrasound sections and accurate hemodynamic data [18, 19].

The 5G remote visualization of critical care ultrasound training systems has better interactivity than traditional methods. The teaching process allows students and teachers to interact in real time, making communication more efficient and convenient. Through the 5G remote interactive platform, the instructor can quickly determine the students’ shortcomings and help them solve specific practical clinical problems. Trainees can also use the platform to communicate their teaching needs directly to the instructor and quickly obtain answers related to their difficulties. The 5G remote interactive platform allows instructors to truly understand the needs of the trainees and their practical abilities promptly adjust the teaching focus according to each trainee’s unique characteristics. This allows the instructor to conduct the relevant educational work in a more targeted manner and further improve the quality of critical care ultrasound training. Our results showed that the experimental group’s theoretical, operational, and total assessment scores were significantly higher than those of the control group. Compared with traditional remote video online meetings, 5G remote visualization technology training system implementation for standardized critical care ultrasound training is more helpful for improving trainees’ teaching and assessment results—particularly in practical skills.

To better understand the effect of the program on actual clinical work, we asked the trainees to rate their levels of satisfaction with the training 1 month after it finished to give them sufficient time to experience the actual value and significance of the course in their clinical work. Results showed that the total satisfaction score of the experimental group was significantly higher than that of the control group. The survey and analysis of the satisfaction indexes further found that compared with the traditional remote video online teaching method, the 5G-based system deepened the trainees’ understanding of the theoretical knowledge underlying critical care ultrasound. Additionally, their practical bedside skills and emergency resilience significantly improved, reducing clinical errors and strengthening their self-confidence.

## Conclusion

By using 5G remote visualization for standardized training and assessment in critical care ultrasound, critical care physicians can more thoroughly understand the theory behind this important clinical skill, better standardize its specific operation and process, and more skillfully apply the technology in clinical practice.

## Data Availability

No datasets were generated or analysed during the current study.
